# Quantitative Analysis Linking Inner Hair Cell Voltage Changes and Postsynaptic Conductance Change: A Modelling Study

**DOI:** 10.1155/2015/626971

**Published:** 2015-01-05

**Authors:** Andreas N. Prokopiou, Emm. M. Drakakis

**Affiliations:** Department of Bioengineering, Imperial College London, Exhibition Road, London SW7 2AZ, UK

## Abstract

This paper presents a computational model which estimates the postsynaptic conductance change of mammalian Type I afferent peripheral process when airborne acoustic waves impact on the tympanic membrane. A model of the human auditory periphery is used to estimate the inner hair cell potential change in response to airborne sound. A generic and tunable topology of the mammalian synaptic ribbon is generated and the voltage dependence of its substructures is used to calculate discrete and probabilistic neurotransmitter vesicle release. Results suggest an almost linear relationship between increasing sound level (in dB SPL) and the postsynaptic conductance for frequencies considered too high for neurons to phase lock with (i.e., a few kHz). Furthermore coordinated vesicle release is shown for up to 300–400 Hz and a mechanism of phase shifting the subharmonic content of a stimulating signal is suggested. Model outputs suggest that strong onset response and highly synchronised multivesicular release rely on compound fusion of ribbon tethered vesicles.

## 1. Introduction

The auditory system provides a link between the mechanical vibrations of our surrounding environment, usually air, and the perception of sound. It consists of two functionally different systems: the auditory periphery and the central auditory neuronal pathways.

The auditory periphery collects sound energy and performs mechanical analysis of incident acoustic waves. The central pathways encode and provide semantic interpretation of the analysed acoustic waves.

The auditory periphery, by means of a cascade of physically distinct tissues which are characterised by different mechanical properties, analyzes sound vibrations. The incoming acoustic waves impinge on the tympanic membrane, which causes a displacement to propagate onto the middle ear ossicles. The ossicles link the tympanic membrane with the oval window, which by being smaller than the tympanic membrane and coupled with the combined lever action of the ossicles perform a mechanical impedance transformation. This transformation allows airborne vibrations to transfer into the perilymph and endolymph in the bony snail-shaped cochlea, where the sound vibrations transpose into fluid pressure differentials. The basilar membrane and Reissner's membrane separate the perilymph and endolymph. Hence sound induced pressure differentials develop on either side of the membranes. This creates displacing forces on the membranes, mainly the basilar membrane, mechanical properties of which vary along its length and thus enable it to resonate preferentially at different frequencies along its length. This is a key feature in the analysis of sound and such tonotopic organisation seems to be a persistent attribute of the central neuronal pathways.

On the surface of the basilar membrane resides the organ of Corti, which consists of a complex arrangement of functionally distinct cells. Two of the cell types residing on the organ of Corti are the outer and inner hair cells. The outer hair cells are considered to provide active control of the basilar membrane vibration. The inner hair cells are considered to be the sensing elements and have afferent neural projections to the central auditory system.

In other words the inner hair cell is the anatomical place where the peripheral auditory processing ends and the central auditory coding begins. The inner hair cell itself is separated into two functional regions. The top half of the cell houses the stereocilia that perform the mechanoelectric transduction of sound at ion gates located near their tip links while the bottom half of the cell perform control of synaptic activity.

The synaptic activity encodes low frequency sounds by maintaining a phase synchronous vesicle release pattern, that is,* phase locking*. For high frequency sounds the frequency of the vesicle release increases. The increase in vesicle release rate combined with the spatial location of excitation, due to the basilar membrane mechanical properties, is referred to as* place coding*. The phase locking response of the neurons is used to compute the inter-aural time difference [1] with a stunning sensory limen of a few microseconds where the onset time of the neuronal response has a prominent role [2].

For the purposes of this study we will focus on the bottom half of the inner hair cell and the structures that facilitate the fine temporal precision in sound encoding. Around ten afferent neurons touch the base of the cell in humans [3, 4] where they form bouton-like chemical synapses. The synapse anatomy consists of a specialised organelle, the* synaptic ribbon*, that is found in the hair cell. The space between the presynaptic hair cell membrane and the postsynaptic neuron membrane is termed as the* synaptic cleft*. In this space neurotransmitter chemical, specifically the chemical glutamate, is released by the hair cell. Glutamate in the synaptic cleft triggers specialised ion channels to open on the postsynaptic neuron cell membrane. These channels are termed as AMPA (*α*-amino-3-hydroxy-5-methyl-4-isoxazolepropionic acid) channels and open in the presence of glutamate and allow the exchange of sodium and potassium ions. As such the release of glutamate in the synaptic cleft by the inner hair cell presynapse via the opening of the AMPA channels on the postsynaptic membrane changes the neuronal membrane conductance. This enables the flux of sodium and potassium ions between the synaptic cleft and the peripheral auditory neuron, causing depolarization and action potential generation. These action potentials are transmitted via saltatory conduction to the spiral ganglion and subsequently continue onto the central auditory pathways.

The synaptic ribbon is the subject of the study presented here. It plays an important role in the synchronisation of sound stimuli and action potential generation as it is thought to mediate the release of the neurotransmitter glutamate. This has been shown through studies where the lack of synapse-anchored ribbons in mutant mice causes reduction in exocytosis and degrades precise action potential onset-timing in the auditory nerve [5]. Furthermore the maturation of the ribbon structure, from a dense bundle to an ellipsoid structure, and its anchoring on the cell membrane marks the onset of hearing in altricial rodents that are born deaf [6].

The synaptic ribbon is not present only in the auditory system. It appears in all sensory cell types which respond to* graded receptor potentials* [7], such as the bipolar photoreceptor cells in the visual system, the vestibular system, and others [6]. Graded receptor potentials are essential in characterising physical quantities over a varying dynamic range. The auditory system is impressive in its performance by having an input dynamic range of around 130 dB SPL. During maturation of the auditory synapse and transition between prehearing and posthearing stage, the transfer function defining the calcium ion influx and vesicle release switches from an exponential relation to a linear one [6, 8, 9]. The linear dependence is also preserved in the postsynaptic current with minimal distortion [10, 11]. This suggests a significant effort in the auditory synapse system to preserve a dynamic range representation by maintaining the graded response instead of a binary on-off response. Furthermore it has been shown that the presence of the synaptic ribbon enhances the statistical representation of graded responses [12].

The synaptic ribbon appears to be a multifunctional device, and there is not a clear understanding of the exact details of its operation [6]. The main suggested function of the synaptic ribbon is its aiding in high rate and sustained neurotransmitter release when excited by long-duration stimulations [13, 14]. It performs this function by tethering neurotransmitter vesicles on its surface thus creating a local store. It is not clear if the synaptic ribbon speeds up or slows down vesicle release, but it is certain that it acts as a mediator to the release of neurotransmitter [15]. Furthermore it is also clear that the morphology of the ribbon synapse defines different populations, or pools, of neurotransmitter vesicles [10, 16]. The vesicles that are located next to the cellular membrane under the ribbon are contributing to the “ready for release” pool, and the vesicles that are tethered onto the synaptic ribbon but are not adjacent to the cellular membrane are contributing to the “reserve pool.” For an illustration see Figure 3.

There is consensus on the fact that the synaptic ribbon aids the neurotransmitter release by colocalizing vesicles and calcium channels [6, 11, 17]. Furthermore it has been suggested that the calcium sensor of the synaptic vesicle is extremely sensitive to calcium channel location especially for weak stimulation with up to threefold change in release probability for a spatial movement of a calcium channel of just 5 nanometers [18]. The spatial colocalization importance can also be verified by the observation that upon maturity and hearing onset of mice, the calcium influx is reduced but the vesicle release increases, whilst the calcium channels migrate in the vicinity of the synaptic ribbon [9]. This spatial reorganization of the synaptic machinery aids in better statistical independence between single, voltage sensitive, and calcium channel opening events and therefore contributes to a higher sensitivity of graded changes of intracellular voltage.

Even though there have been significant developments in high resolution imaging and electrophysiological approaches, several of the elementary characteristics of the ribbon synapse remain uncharacterised. Genetic and chemical approaches to identify key molecules in the vesicular mechanism seem to be hindered by the functional redundancy of the vesicle tethering and release complexes [6]. Furthermore much of what is known so far is based on induced depolarization of the inner hair cells and not via the graded response of the stereocilia residing on the top of the cell.

The scope of the model described in this paper is to simulate stochastic neurotransmitter vesicle release events resulting from acoustic waves arriving at the tympanic membrane and hence estimate the postsynaptic conductance change of the auditory nerve-fibre terminus.

Synaptic ribbon morphology variations [19–21] indicate that the electrical behaviour of the afferent auditory neurons is shaped by both pre- and postsynaptic elements. Furthermore the adaptation of the neuron response to stimulation has been shown to be mainly determined by the vesicle release machinery [10]. The synaptic elements that shape the electrical behaviour of the auditory synapse have not yet been integrated in an explicit model [6]. The work presented here integrates previous studies in a model which reproduces experimentally observed phenomena and presents some novel simulation data on vesicle release patterns in response to sound stimuli.

## 2. Model/Methods

**Figure 1:**
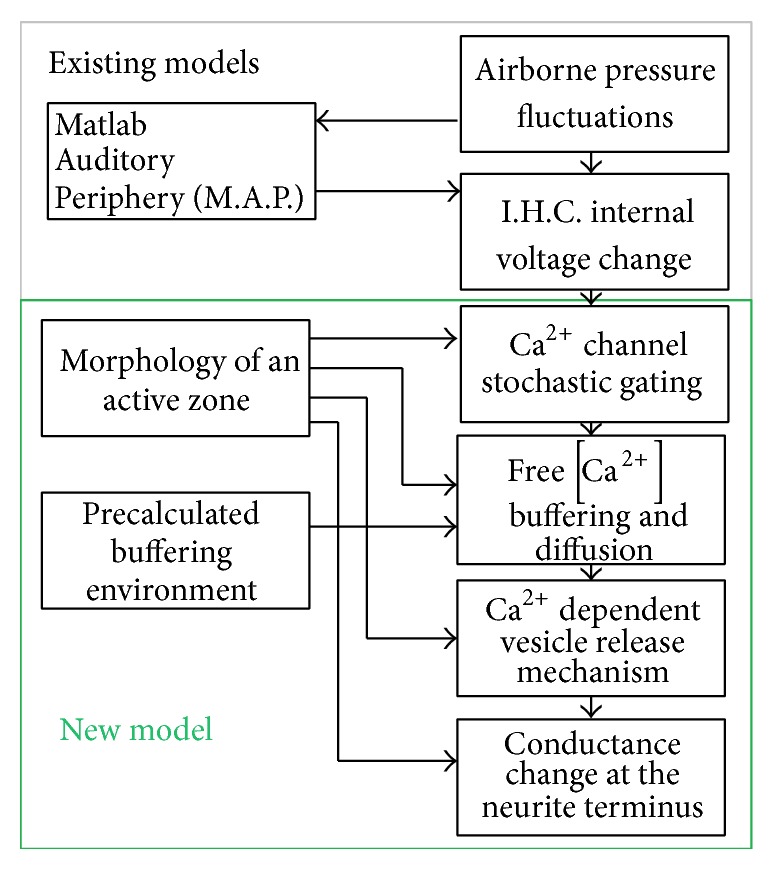
Flowchart illustrates the top level organisation and the modular structure of each “submodel” as the union of preexisting models and the new model developed and presented here. The overall cascade of models represents the physiological paradigm of action potential generation in the auditory synapse. Matlab Auditory Periphery and the modelling of sound to intracellular voltage are described elsewhere [22, 23].

The model used to estimate the vesicle release is a novel realisation of the ribbon synapse, amalgamated with an existing model of the auditory periphery; see Figure 1. The preexisting model of the auditory periphery that is used for the calculation of the internal voltage change of the inner hair cell in response to a sound wave is termed M.A.P. (Matlab Auditory Periphery) and is freely distributed [22]. M.A.P. cascades physiological stages that lead to the resolution of the voltage changes within the inner hair cell. It models the head transfer function and the tympanic membrane motion with the acoustic reflex. Moving into the middle-inner ear it models the ossicular chain vibrations and the basilar membrane motion and considers the olivocochlear efferent feedback effect to model the effect of outer hair cell action. Motion of the basilar membrane causes shear stress to develop on the stereocilia of the inner hair cells, which causes them to displace. Their displacement triggers the mechanoelectric transduction mechanism at the stereocilia tips on the top of the inner hair cell that generates an internal voltage change, which is also modelled by M.A.P. (the interested reader may consult [23]).

The new model of the auditory synapse consists of two different sections: (1) the morphological setup of the ribbon synapse geometry, discussed in Section 3 and (2) the temporal evolution of the virtual organelles and their interactions, discussed in Section 4.

## 3. Morphology

The active zone and the synaptic ribbon spatial topology is calculated as the analytical solution to certain geometric constraints. This section is divided into three subsections that deal with the details of the model implementation.  Section 3.1, termed “Topology of an Active Zone,” describes the population of the presynaptic cell membrane with voltage sensitive calcium channels.  Section 3.2, termed “Morphology of a Ribbon Synapse,” reports the geometric parameters that the model uses to place a synaptic ribbon in a three-dimensional volume floating above the presynaptic cell membrane.  Section 3.3, termed “Neurotransmitter Vesicle Space Population,” describes how the model populates the three-dimensional volume above the presynaptic cell membrane with neurotransmitter filled vesicles.

### 3.1. Topology of an Active Zone

**Figure 2:**
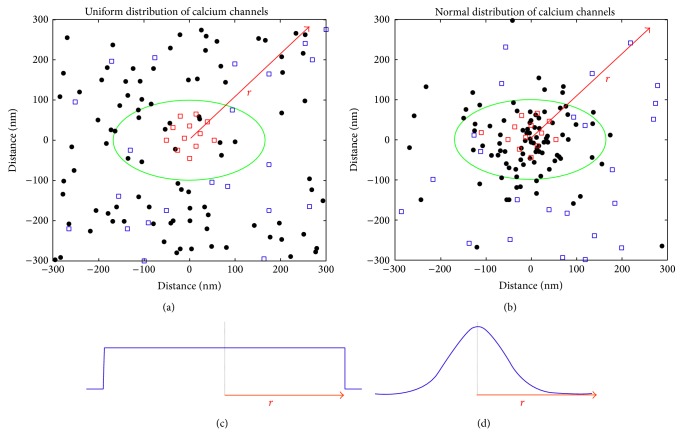
(a) and (b) show a top view of the active zone which has roughly 90 calcium channels. The synaptic ribbon extent is outlined by the green line as an oval circle, the filled circles represent calcium channels, and the open circles represent synaptic vesicles (red signifies ribbon tethered vesicles, whereas blue signifies freely floating vesicles). The line *r* indicates the radius of the active zone. (a) illustrates an immature synapse characterised by the uniform distribution spread of calcium channels shown in (c). (b) illustrates a mature synapse characterised by the normal distribution spread of calcium channels with the peak of distribution being the centre of the synapse shown in (d). (c) and (d) show the probability distribution of channel placement along the active zone diameter. (c) is a uniform distribution limited to the extent of the active zone diameter. (d) is a normal distribution with a standard deviation (SD) equal to one-fourth of the radius, that is, *r*/4. Note that the selection of SD = *r*/4 is a conjecture based on visual inspection and descriptions of the maturation of the synapse [6, 9, 26].

The active zone consists of the two-dimensional distribution of calcium channels on the presynaptic area and a thicker, bow-shaped, postsynaptic density [6]. In this model only the presynaptic active zone is considered. Parameters used in the estimation of the active zone include (1) its radius, (2) a mean calcium channel number, (3) normal or uniform spatial distribution of the channels from a central axis, and (4) a conjectured calcium channel diameter; see Figure 2 for an example.

The calcium channels are randomly placed within a circle defined by the radius of the active zone. If the placement of the calcium channels is assumed to vary with a uniform distribution, then the calcium channels are evenly spread out within the active zone circle since every possible location has the same possibility of channel placement. This results in an equal density of the calcium channels on the presynaptic membrane which reflects the immature prehearing state of the active zone [9]. If the placement of the calcium channels is assumed to vary with a normal distribution, the mean of the distribution is aligned with the centre of the presynaptic membrane; see Figure 2. This results in a higher density of calcium channels under the ribbon synapse and this colocalization of voltage sensitive calcium channels and synaptic ribbon is thought to be part of the maturing process of the auditory synapse [6, 9]. The dimensions used for the simulations presented here are given in Table 1.

### 3.2. Morphology of a Ribbon Synapse

**Figure 3:**
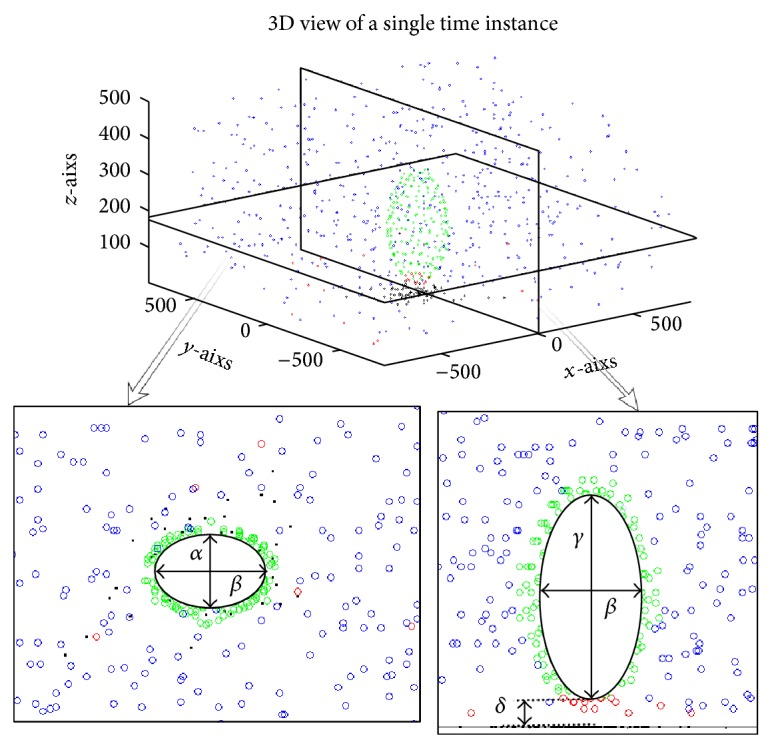
Top and side cross-sectional views of an ellipsoid ribbon synapse. The parameters that describe the shape are *α* = *x* axis radius, *β* = *y* axis radius, and *γ* = *z* axis radius and *δ* is the separation distance from the presynaptic membrane. The circles indicate neurotransmitter vesicles; the closely packed to the synaptic ribbon green vesicles are considered tethered to the ribbon synapse and make the “ribbon associated” pool, the red vesicles close to the presynaptic membrane make the “ready for release” pool, and the blue vesicles that are haphazardly spread in the surrounding volume make the “free floating” pool.

The synaptic ribbon for the mammalian auditory synapse is reported to progress from an electron dense bundle to an enlarged oval shape and attach to the active zone via a single link [6]. The parameters used in the estimation of the three-dimensional synaptic ribbon are the three axes of the ellipsoid representing its height, its width, and its depth; see Figure 3 for an illustration. The model is implemented by placing the synaptic ribbon in the centre of the simulated volume, at a preset distance, *δ* in Figure 3, from the cellular membrane and occupying the space defined by the triaxial ellipse as shown in Figure 3. Note that the origin point of the active zone and the main axis of the ellipsoid are aligned (see Figure 2) which places the synaptic ribbon above the centre of the active zone. The dimensions used for the simulations presented here are given in Table 1. For a few examples of various morphologies see Figure 11.

### 3.3. Neurotransmitter Vesicle Space Population

The neurotransmitter vesicles are floating in the intracellular space and when they come in close proximity to the synaptic ribbon they form a temporary tether which is not fully understood yet [6, 13]. The vesicles are considered, via random Brownian motion, to refill certain repositories: the “ready for release” pool, the “ribbon associated” pool, and the “free floating” pool. This is modelled by placing the vesicles in the simulation volume by choosing a random set of three-dimensional coordinates. The rules that are set by the algorithm are that the certain coordinates do not cause vesicles to overlap with each other, nor the ribbon synapse. Furthermore the space surrounding the synaptic ribbon is completely filled with vesicles until no other vesicle can fit without overlapping with an existing one. The parameter characterising the synaptic vesicles is their diameter, which is set at a mean diameter of 35 nm and a standard deviation of 10 nm; see Figure 10(b) for a vesicle diameter distribution.

The neurotransmitter vesicle occupancies that are immediately adjacent to the synaptic ribbon are considered to populate the tethered, or “ribbon associated” pool. Vesicle occupancies that have a distance from their outer wall to the cellular membrane that is less than 15 nm are considered to populate the “ready for release” pool. This range of distances, up to 15 nm, is considered to be the limit for the adhesion event that creates the fusion between the vesicle and the presynaptic membrane that can lead to exocytosis [24]. The remaining vesicle occupancies compose the “reserve” (or freely floating) vesicle pool. For an example of the population of vesicles within the simulated volume see Figure 3.

## 4. Chronology

The temporal events involved in inner hair cell processing are separated into six different sections and the driving parameter for all sections is the internal voltage of the inner hair cell: (1) The electrochemical calcium current is calculated, (2) the calcium channels “open”/“close” states are computed, (3) calcium spatio-temporal buffering is estimated, (4) the calcium concentration change for the duration of the stimulation is calculated for the “ready for release” vesicle occupancies, (5) the compound vesicle release pattern is calculated and finally (6) the excitatory post synaptic conductance is calculated. The sampling rate of all the signals processed is at 44.1 kHz and an up-sampling at 10 MHz was performed for Monte Carlo runs. The up-sampling is to ensure that the sampling rate in the Monte Carlo process, which is notorious for being sensitive to sampling time periods, did not alter the estimated dynamics. During the development stage of the model the Monte Carlo sampling rate was determined by an iterative process by which the input was up-sampled until the computed output was undistinguishable between the chosen sampling rate and higher sampling rates.

The only model elements that are assumed to be moving during the simulation period are the free neurotransmitter vesicles. Contrary the synaptic ribbon, the calcium channels and the tethered neurotransmitter vesicles are assumed to be static. The free movement of neurotransmitter vesicles is simulated by randomly filling the volume with all possible occupancies of neurotransmitter vesicles. At any time a fraction of those locations is considered populated. This percentage is calculated so as to relate to the vesicle density to correspond to a realistic amount of neurotransmitter vesicles. At the next time instant a different set of predefined locations become populated thus simulating the random motion of the vesicles within the simulation volume. This becomes particularly important for untethered vesicles floating next to the cellular membrane. At that specific location the vesicle's travel time will define whether the vesicle will bind to the membrane and release its content to the synaptic cleft or not. For the purposes of this study vesicles that are next to the membrane and are not tethered to the synaptic ribbon were allocated their occupancy for 12 ms. Such a value was chosen because it is the calculated travel time for the maximum distance the vesicle can fuse with the cellular membrane [24, 25].

The remaining section is divided into seven subsections: (1) Electrochemical calcium current flux, (2) Ca channel dynamics, (3) Calcium chemical buffering, (4) Calcium concentration estimation, (5) Vesicle release mechanism, (6) Compound fusion release, (7) Excitatory postsynaptic conductance estimation.

### 4.1. Electrochemical Calcium Current Flux

When a calcium channel opens there is an influx of calcium ions in the cell, altering the local calcium ion concentration at the base of the inner hair cell. This flux is driven by both an electric and a chemical gradient. In this model the chemical concentration of the bulk is assumed to remain unchanged by a calcium channel opening event. Therefore a constant baseline calcium ion, chemical-gradient driven flux of −0.2 pA, is maintained in the case of a channel opening. The electrical drive is modelled by replacing the calcium channel by a resistor with a conductance value of 14.7 pS [9, 26].

### 4.2. Ca Channel Dynamics

Estimating the calcium channel “open”/“close” dynamics takes place in two separate steps as dictated by the model codified by (1). First the kinetic rates are calculated. The next step is to use the kinetic rates to calculate the probability of state (“open” or “close”) transition by means of a Monte Carlo model. The Gilespie algorithm was attempted for faster computation but was found to be good only at estimating the steady state solution.

**Figure 4:**
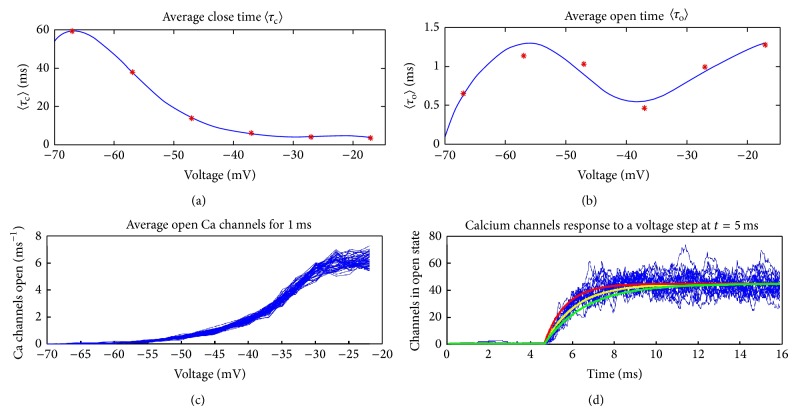
(a) The average “close” time, *τ*_*c*_, and (b) the average “open” time, *τ*_*o*_, of a single calcium channel with respect to cell membrane voltage. A fourth-order polynomial fit was used to extrapolate recorded results (shown in red stars) from previous studies [9, 26]. Plot (c) shows the rate of Ca^2+^ channels opening given a certain voltage. Plot (d) shows many repetitions of the response of a collection of calcium channels given a step increase in intracellular voltage from −65 mV to −20 mV lasting 100 ms. The rise time of this ensemble was found to be between 2 ms (red line) and 1 ms (green line). Both results agree with a rise time of 1.4 ms [9] which is represented by the yellow line.

First the kinetic rates *k* ^+^(*t*) and *k* ^−^(*t*) are calculated from the average open (*τ*_*o*_(*V*)) and the average “close” (*τ*_*c*_(*V*)) time, respectively, as shown in (1); note that *V*(*t*) denotes the intracellular voltage. The voltage dependence of the “open” and the “closed” times is calculated using a fourth-order polynomial fit that was used to extrapolate recorded results; see Figure 4. Consider

(1) $$\begin{array}{c} C_{0}\underset{k^{-}\left(t\right)}{\overset{2k^{+}\left(t\right)}{⇌}}C_{1}\underset{2k^{-}\left(t\right)}{\overset{k^{+}\left(t\right)}{⇌}}\text{Open},\\ k^{+}\left(t\right)=\frac{1}{\tau_{o}\left(V\left(t\right)\right)},\;\;k^{-}\left(t\right)=\frac{1}{\tau_{c}\left(V\left(t\right)\right)}. \end{array}$$

The second step, after the kinetic rate estimation, is to calculate the probabilities of state transitions [27]. A three-state model using two “closed” and one “open” state was used, as shown in (1). A two-state and a four-state model were also studied. The two-state model caused excessive vesicle release—much faster than the reported 1.4 vesicles/ms/ribbon [14]. The four-state model exhibited very slow rise times to step voltage stimuli for calcium channel opening—much slower than the reported 1.4 ms [9]. For these reasons this study employs the three-state model in (1).

Calcium channel state transitions were calculated by means of a time-dependent matrix *Q*; see (3). The probability of state change is calculated as shown in

(2) $$\begin{array}{c} \vec{P}\left(t+n\Delta t\right)=Q{\left(t\right)}^{n}\vec{P}\left(t\right), \end{array}$$

where $\vec{P}$ denotes a vector signifying the state of the calcium channel, Δ*t* denotes the sampling time of the simulation, and *n* denotes how many samples ahead we simulate (for this study every time sample was simulated; therefore *n* = 1). Since we employ the three-state model of (1), $\vec{P}=[1,0,0]$ signifies that the channel is at state *C*_0_ and $\vec{P}=[0,1,0]$ signifies state *C*_1_; thus the channel is closed for both. When $\vec{P}=[0,0,1]$ the channel state corresponds to “open.” Note that only one state is possible at any given time:

(3) Qt=1−2k+tΔtk−tΔt02k+tΔt1−k−tΔt−k+tΔt2k−tΔt0k+tΔt1−2k−tΔt.

For an ensemble of a few hundred calcium channels both the average rise time and the average number of open channels agree with published results [9, 26] (see Figure 4).

### 4.3. Calcium Chemical Buffering

The extent to which the free calcium ions reach in the intracellular space is very limited [28–30]. Modelling studies have shown that a calcium ion concentration capable of releasing vesicles exists in the vicinity (10–100 nm [31, 32]) of the mouth of the calcium channel when it opens. Furthermore the endogenous buffering environment ensures the quick absorption of free calcium ions so that a residual built-up of local calcium concentration is not created [33]. Further studies using different calcium buffers such as EGTA, ATP, and BAPTA confirm the aforementioned modelling studies [16, 30].

**Figure 5:**
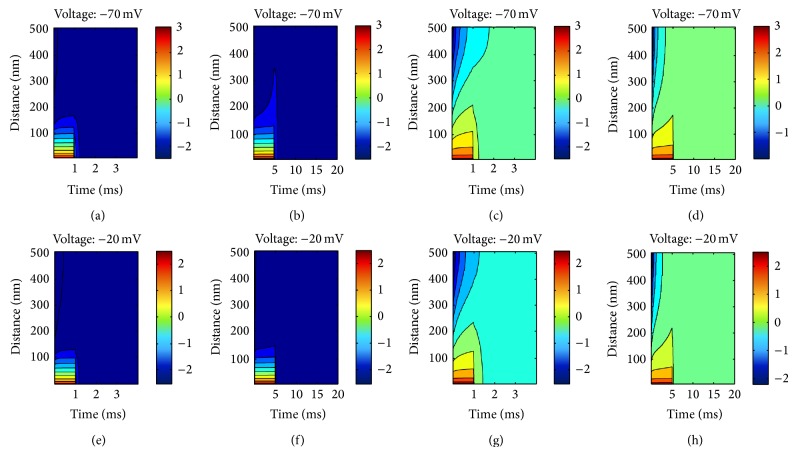
Spatiotemporal calcium evolution as calcium channels open. The plots show the base 10 logarithm of the intracellular calcium ion concentration as it varies with distance around the mouth of the channel and as time passes. Note that the calcium ion spread inside the hair cell depends strongly on three factors: (1) the buffering environment ((a), (b), (e), and (f) correspond to BAPTA, while (c), (d), (g), and (h) correspond to endogenous buffers) (2) the voltage across the channel ((a), (b), (c), and (d) correspond to −70 mV across the cell membrane while (e), (f), (g), and (h) correspond to −20 mV across) (3) the time the channel remains open (the channel in (a), (c), (e), and (g) stays open for 1 ms while in (b), (d), (f), and (h) it stays open for 5 ms). For the buffering chemical constants consult Table 2.

The calcium buffers EGTA, ATP, and BAPTA have different length-constants, which means that calcium ions will travel different distances from the mouth of the channel depending on the combinations of said buffers. For model verification purposes the effect of various combinations of EGTA, ATP, and BAPTA and endogenous buffers was modelled. The buffering calculation is performed using CalC [34]; see Figure 5 for some exemplar results. The chemical constants that characterise each buffer species used in this model are shown in Table 2.

The spatiotemporal buffer calculation for each calcium channel opening on the presynaptic membrane is very computationally intensive and makes the implementation of the model practically unrealisable. To work around this problem a buffering “environment” is created which is based on the sampling rate of the input signal, 44.1 kHz in all model outputs shown here. The “environment” is a look-up table which contains precomputed values of the possible voltage levels attainable within the inner hair cell, ranging between −70 mV and −17 mV, and possible open times of the calcium channels with a maximum limit of 10 ms of continuous open time. Some possible combinations of open time and across membrane voltage are shown in Figure 5. The look-up table is incrementing time in steps equal to one over the sampling rate; hence every calcium channel opening event can be exactly accommodated by this table without the need to round up or down to a preset of selected values. Furthermore the look-up table is incrementing with steps of 0.1 pA. This value was selected based on the observation during the development of the model that any change smaller than 0.1 pA creates a negligible change in the internal calcium concentration surrounding the mouth of the channel.

### 4.4. Calcium Concentration Estimation

**Figure 6:**
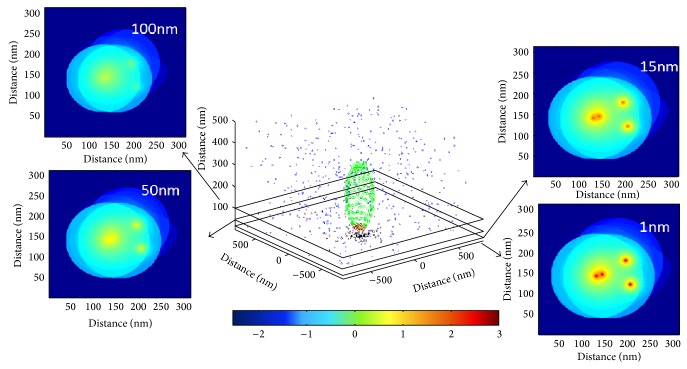
Calcium ion concentration built-up from the calcium channels opening and signifying a practically concurrent opening of four calcium channels. Inserts show top view cross-sections at different *z*-axis distances exemplifying the short spatial extent of calcium ion reach around the channels mouth. The inserts show the base 10 logarithm of the internal calcium ion concentration with a colour coding as indicated by the colour bar in the diagram.

Following the generation of the buffering “environment” (see previous text) the calculation for the calcium ion influx surrounding the channel mouth is performed separately for each channel, but the overall internal calcium ion concentration contribution at each vesicle sensor is derived from a linear summation of calcium channels ion influx. For an illustrative situation see Figure 6. The linear summation of the calcium channels is possible under the assumption that there is no coupling between calcium channels. This assumption is justified on the basis of the nanodomain coupling that is thought to exist between the calcium channels and the vesicular sensors in the auditory synapse [16, 30]. Calcium channel coupling occurs for other behaviours such as a microdomain or an oscillation behaviour by utilizing calcium induced calcium release mechanisms which do not exist in nanodomain synapse situations [35]. In other words, since nanodomain coupling between sensor and calcium channels exists linear summation of each channels ion influx is possible [31].

For every “ready for release” vesicle occupancy the opening of neighbouring calcium channels increases the internal calcium ion concentration picked up by its “calcium sensor,” which varies as a function of time, [Ca^2+^](*t*). The parameters that affect the internal calcium ion concentration estimation at the vesicle are the distance from calcium channels, the channel “open” time, the current flow (i.e., the internal voltage), the buffering environment, and the external calcium ion concentration. For every individual neurotransmitter vesicle a characteristic [Ca^2+^](*t*) is calculated in response to an intracellular voltage change, *V*(*t*).

### 4.5. Vesicle Release Mechanism

The neurotransmitter vesicle fusion with the cellular membrane followed by the release of glutamate is considered to rely on the cooperativity of five calcium ions [36, 37] and is modelled by means of (4). Note that for presentation purposes [Ca^2+^](*t*)≡[Ca]:

(4) B0⇌Ca5kon⁡koff⁡B1⇌Ca4kon⁡2koff⁡bB2⇌Ca3kon⁡3koff⁡b2B3⇌Ca2kon⁡4koff⁡b3B4⇌Cakon⁡5koff⁡b4B5⟶γfused.

The postulated “calcium sensor” of the neurotransmitter vesicle is symbolized in (4) with *B*_*n*_ where *n* indicates the bound calcium ions on the sensor. The dynamics of the equation were determined in [37] and are as follows: *b* = 0.4, *k*_*on*⁡_ = 27.6 *μ*Ms^−1^, *k*_*off*⁡_ = 2150 s^−1^, and *γ* = 1695 s^−1^. The vesicle release system is solved using a five-state Monte Carlo with a last deterministic step. The state transition vector ${\vec{P}}_{B}$ is calculated using (5). The vesicle's “calcium sensor” probability for state transitions was calculated by means of a calcium-depended matrix *H* shown in (6). Note that Δ*t* is equal to the sampling time of the simulation and the set of equations shown in (7)–(12) are the values of the diagonal of matrix *H* as shown in (6).

The vector ${\vec{P}}_{B}$ is a six-element array where all values are zero, except one which symbolizes the current state of each vesicle occupancy in the “ready for release” pool. For example, in the case where no calcium ions are bound ${\vec{P}}_{B}$ is at its lowest state; that is, ${\vec{P}}_{B}=[1,0,0,0,0,0]$. The binding of a single calcium ion to the “calcium sensor” will cause the ground state to be evacuated and the next state to be occupied; that is, ${\vec{P}}_{B}=[0,1,0,0,0,0]$. Note that only one state is possible at any given time; see Figure 9(c) for an example of a time evolving state transition. The last deterministic step models the delay in the conformation change of the proteins forming the membrane fusing complex. Having all five calcium ions bound on the sensor does not guarantee vesicle release, since when a calcium ion detaches from the sensing mechanism before completion of fusion, the complex goes back to the not-fused state; an example of this is illustrated in the insert of Figure 9(c). The delay of the protein conformation is modelled by characterising a vesicle release event if the binding of five calcium ions (e.g., state ${\vec{P}}_{B}=[0,0,0,0,0,1]$) is maintained for a period of time *τ*_*γ*_ = 1/*γ*:

(5) $$\begin{array}{c} {\vec{P}}_{B}\left(t+\Delta t\right)=H\left(t\right){\vec{P}}_{B}\left(t\right), \end{array}$$

(6) $$\begin{array}{c} H\left(t\right)=\left(\begin{array}{cccccc} D_{0} & k_{\mathrm{off}}\left(t\right)\Delta t & 0 & 0 & 0 & 0\\ 5k_{\mathrm{on}}\left[\text{Ca}\right]\Delta t & D_{1} & 2k_{\mathrm{off}}b\Delta t & 0 & 0 & 0\\ 0 & 4k_{\mathrm{on}}\left[\text{Ca}\right]\Delta t & D_{2} & 3k_{\mathrm{off}}b^{2}\Delta t & 0 & 0\\ 0 & 0 & 3k_{\mathrm{on}}\left[\text{Ca}\right]\Delta t & D_{3} & 4k_{\mathrm{off}}b^{3}\Delta t & 0\\ 0 & 0 & 0 & 2k_{\mathrm{on}}\left[\text{Ca}\right]\Delta t & D_{4} & 5k_{\mathrm{off}}b^{4}\Delta t\\ 0 & 0 & 0 & 0 & k_{\mathrm{on}}\left[\text{Ca}\right]\Delta t & D_{5} \end{array}\right), \end{array}$$

(7) D0=1−5kon⁡Ca2+tΔt,

(8) D1=1−koff⁡tΔt−4kon⁡Ca2+tΔt,

(9) D2=1−2koff⁡bΔt−3kon⁡Ca2+tΔt,

(10) D3=1−3koff⁡b2Δt−2kon⁡Ca2+tΔt,

(11) D4=1−4koff⁡b3Δt−kon⁡Ca2+tΔt,

(12) D5=1−5koff⁡b4Δt.

It has been reported that vesicles reappeared on the synaptic ribbon almost as rapidly as they were released [38] so no reduction in the number of vesicles was assumed. For some exemplar results of vesicle “calcium sensor” state transitions because of calcium channels' opening and closing dynamics see Figure 9.

### 4.6. Compound Fusion Vesicle Release

The role of the synaptic ribbon is thought to be a harbour for neurotransmitter filled vesicles that brings them to close proximity to the auditory synapse [6]. The exact mechanism of transport of the ribbon tethered vesicles to the release site is not clear [13, 39] but evidence indicates that compound fusion occurs at the ribbon synapse [40]. Compound fusion is the process by which synaptic vesicles fuse to form a larger vesicle that is held in place with the synaptic ribbon tethers. This process is modelled by using the hypothesis that the longer the next-to-membrane vesicle remains unreleased the more the compound fusion events with adjacent vesicles happen that increase its size. This means that when the vesicle does release its contents the entire fused collection of vesicles gets released, and the process restarts from a single vesicle.

**Figure 7:**
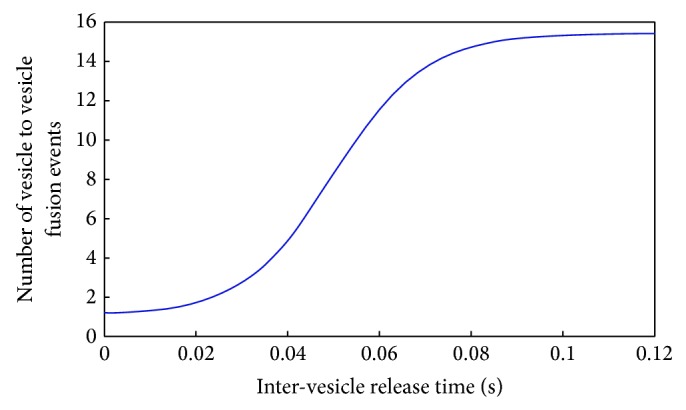
The relation between inter-vesicle release time and compound fusion.

**Figure 8:**
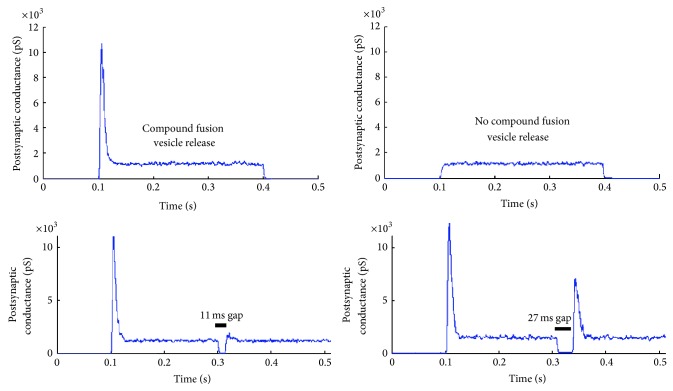
Examples of the strong onset response elicited by compound fusion. The results show the averaged postsynaptic conductance change following 100 iterations of a step change in the intracellular voltage from −70 mV to −20 mV. Taking away the compound fusion mechanism no onset response is observed. Forward masking is examined by introducing gaps in stimulation to observe recovery of response.

The process is modelled as a first order Boltzmann function shown in (13) and in Figure 7 where IVT is the inter-vesicle release time, which is a measure of how long the synaptic vesicle remains next to the cell membrane but is not released, IVT_half_ = 30–50 ms is the inter-vesicle release time at which half of the maximum possible vesicles fuse, *S* = 0.005 ms^−1^ is the sensitivity of fusion events to inter-vesicle release time, and finally FV_max⁡_ is the maximum possible fused vesicles. Note that FV_max⁡_ is calculated based on the morphometric properties of the ribbon synapse, specifically FV_max⁡_ = ribbon  associated  vesicles/ready  for  release  vesicles. Furthermore the compound fusion dynamics are illustrated in Figure 7 where a limiting value of one vesicle signifies no compound fusion events take place before release and the asymptotic maximum value signifies the maximum compound fusion possible as it is constraint by the ribbon shape. Note that the values chosen for IVT_half_ and *S* are conjectures based on the “good” model output shown in Figure 8:

(13) $$\begin{array}{c} \text{Fused}\;\;\text{Vesicles}=\frac{{\text{FV}}_{\max}-1}{1+\exp\left(\left({\text{IVT}}_{\text{half}}-\text{IVT}\right)/S\right)}. \end{array}$$

### 4.7. Excitatory Postsynaptic Conductance Estimation

After the fusion of the neurotransmitter vesicle with the cellular membrane, glutamate is released in the synaptic cleft. This will activate AMPA receptors on the postsynaptic neuron membrane and open ionic channels, thus changing the neuron membrane conductance. This causes an influx of ions (sodium and potassium) in the neuron terminus and when a threshold is exceeded an action potential is generated.

The parameters that affect the postsynaptic conductance value *G*(*t*) are the number of vesicles released and their respective volume, how many AMPA channels an average synaptic vesicle opens and the average “open” time of an AMPA channel. This is modelled using (14), which represents the convolution of the AMPA channel alpha function with the open channels for every given time instant, scaled by the conductance value per AMPA channel:

(14) $$\begin{array}{c} G\left(t\right)=\epsilon \left[\frac{t}{\tau_{\alpha }}e^{-t/\tau_{\alpha }}*\overset{M\left(t\right)}{\underset{i=1}{\sum }}{\left(\frac{D_{\text{ves}}\left(i,t\right)}{D_{\text{avg}}}\right)}^{3}\beta \right]. \end{array}$$

**Figure 9:**
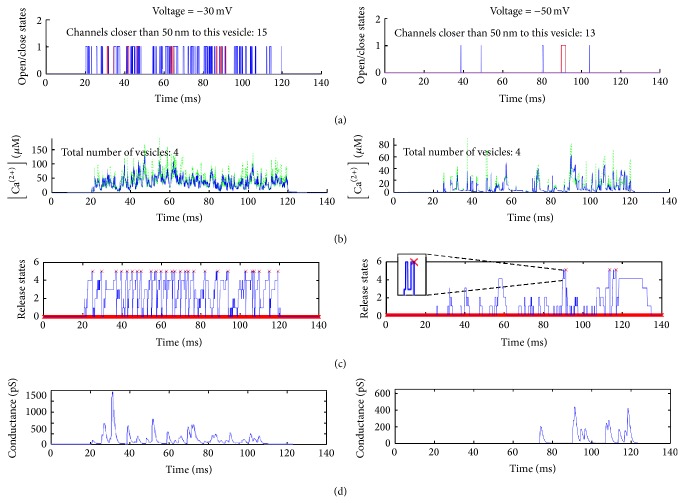
The temporal response of the voltage dependent structures of the synapse anatomy resulting in a change in the postsynaptic conductance. The stimulating signal used is a 100 ms depolarising pulse of −30 mV (left column) and −50 mV (right column). (a) An illustration is shown of the superimposed opening and closing responses of a selection of a few calcium channels that are within a 50 nm radius around a randomly selected position just under the synaptic ribbon. The distance of 50 nm was chosen for clarity of the figure. Note that the most active channel is shown in red. (b) The free calcium ion concentration at the postulated “calcium sensor” of some of the synaptic vesicles that are ready for release. In blue the vesicle whose pattern of release is shown in the next panel. In green a selection of a few of its neighbouring vesicles, thus showing the differences of spatial allocation in calcium sensing. (c) The vesicle release mechanism discrete steps are shown with a red “×” marking the release events. Note that having five Ca^2+^ bound onto the exocytotic mechanism does not always guarantee release (insert). (d) The aggregated response from the release of the synaptic vesicles is changing the postsynaptic conductance in response to the internal cell being depolarised.

**Figure 10:**
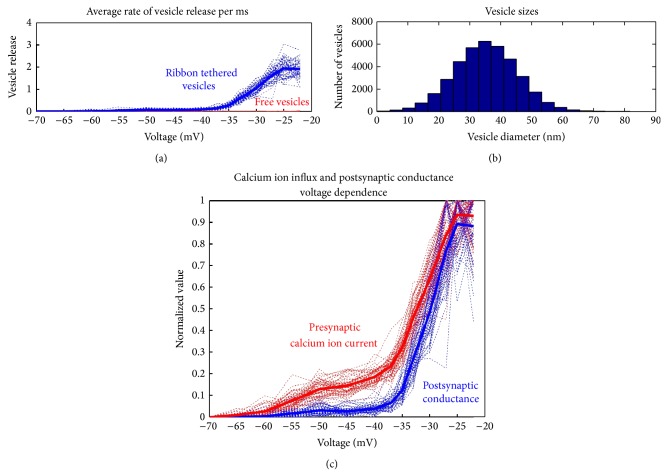
Presynaptic calcium and postsynaptic conductance voltage dependence. (a) Average rate of vesicle release (vesicles per millisecond) when presented with a 100 ms depolarising voltage pulse of varying strength. Note that the blue lines represent ribbon tethered vesicles while the red lines represent free floating vesicles being released. Clearly the presence of the synaptic ribbon facilitates an almost linear release of vesicles and thus the ability to separate different voltage levels. (b) A histogram of all released vesicles' diameters during this analysis. The model randomises the diameter of a released vesicle by fitting a normal distribution with a mean diameter of 35 nm and a SD of 10 nm [41, 56]. (c) The superimposed result of a number of simulation runs (between 30 and 40 repetitions) where 100 ms depolarising voltage pulses of varying strength were used. The red line shows the normalised presynaptic calcium current, while the blue line shows the normalised postsynaptic conductance change. Both agree with published results [11, 52, 57].

The quantity *ϵ* = 20 pS denotes the conductance change of the postsynaptic membrane per AMPA channel opening [41], *τ*_*α*_ = 0.59 ms is the time constant of the AMPA channel [41], *β* = 30 is the average receptors opening following an exocytosed vesicle [41], *M*(*t*) is the total vesicle release events per time instant, *D*_ves_ is the individual channel diameter (see Figure 10), and finally *D*_avg_ = 35 nm corresponds to the average synaptic vesicle diameter [41]. For some exemplar results of postsynaptic conductance change because of neurotransmitter vesicle release see Figure 9.

As are shown in Figure 1 the morphology estimations and the chemical buffering are assumed unaffected by the airborne pressure fluctuations. In Section 3 and in Section 4.3 all the parameters used to define the models, specifically Tables 1 and 2, are not time variant. This fact was exploited for code optimization by generating a precalculated environment, which is utilised as some form of look-up table with parameters that are unique for the specific geometry and chemical environment of the model. These parameters control the calculations in the calcium channel stochastic “open”/“close” dynamics, the calculation of the calcium ion concentration detected by the vesicle's “calcium sensor,” and finally the calculation of the postsynaptic conductance at the afferent neuron in response to airborne sound fluctuations. An example that summarises the dynamic signals of the model is shown in Figure 9.

## 5. Model Outcomes

The study presented here addresses two main questions. First, what is the role of the synaptic ribbon and how does its action and morphology affect the output of the sensory cell? Second, how does the synapse itself contribute to phase locking?

### 5.1. Synaptic Ribbon Shape and Resulting Dynamics

The role of the synaptic ribbon as a mediator to vesicle release is exemplified in Figure 10(a) where it is explicitly shown that it creates an almost linear response to changing intracellular voltage and vesicle release events. The behaviour is practically absent for the freely floating vesicles. As a consequence of the almost linear dependence of the vesicle release on intracellular voltage the almost linear behaviour of the postsynaptic conductance is observed in Figure 10(c), following the presynaptic calcium ion influx. Repeated simulation runs indicate that the overall trend of the postsynaptic conductance in response to sound can be inferred from a collection of a few synapse responses (see Figure 10) which agrees well with the physiological number of synapses attached to a single inner hair cell.

**Figure 11:**
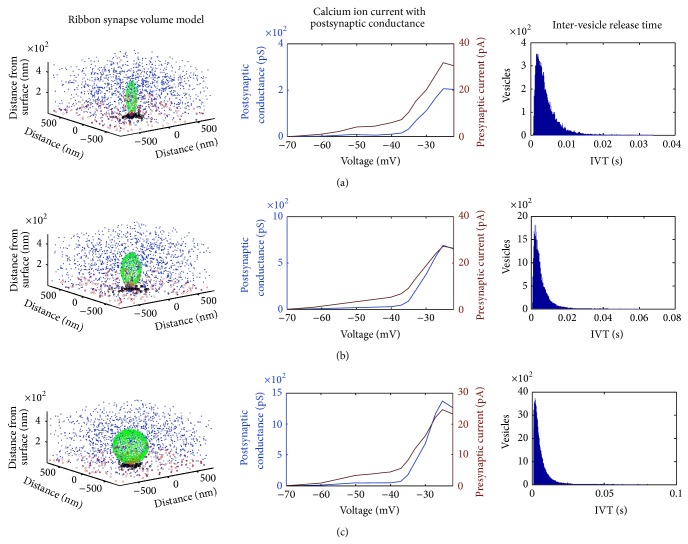
Row (a) illustrates the results from a disk shaped synaptic ribbon resembling a mammalian photoreceptor synapse and row (b) shows the simulations from an ellipsoid shaped synaptic ribbon resembling a mammalian auditory synapse while the third row (c) depicts the results from a sphere shaped ribbon with dimensions taken from those obtained from an amphibian papilla. For a list of numerical parameters see Table 1. The first column graphically displays the locations of all synaptic neurotransmitter vesicles in the model; blue marks the freely floating vesicles and green marks the ribbon tethered vesicles while red marks the vesicles that are free for release given their proximity to the membrane; the calcium channels are indicated with black dots. The second column plots together the presynaptic calcium ion influx (red) and the corresponding postsynaptic conductance of the opposite auditory nerve neurite (blue). The *x*-axis indicates the variation in intensity of the 100 ms voltage pulses used to excite the synapse. The third column is a histogram of the inter-vesicle release time intervals (IVT) of all the vesicle releases. All histograms exhibit a peak at roughly 2.3 ms. In other words, when the synapse is fully driven by a step change in the intracellular voltage, a neurotransmitter filled vesicle will tend to be released at around 430 Hz.

Another main factor in vesicle release is the variance of the ribbon morphology. A novel comparison has therefore been made where the shape of the ribbon varies from a flat disk, more common in retinal photoreceptor [42], to an ellipsoid, more common in mammalian inner hair ribbon synapses [43] and to a sphere of increased radius found in the amphibian papilla [44] (see Figure 11). For all three ribbon morphologies the presynaptic calcium current was the same, on average, since the calcium channel number was not changed. Even though the calcium current was the same it was observed that the average postsynaptic conductance increases with increasing volume of the synaptic ribbon; see Figure 11. The increased surface area above the active zone harbours more neurotransmitter vesicles and therefore permits a bigger volley of neurotransmitter vesicles to be released. This indicates a more potent vesicle release mechanism, which is more responsive to graded intracellular voltage fluctuations. However as the inter-vesicle release time histograms of Figure 11 suggest the rate of release of vesicles is unaffected by the shape of the synaptic ribbon. This is not very surprising since the vesicle release dynamics were identical for all three shapes.

**Figure 12:**
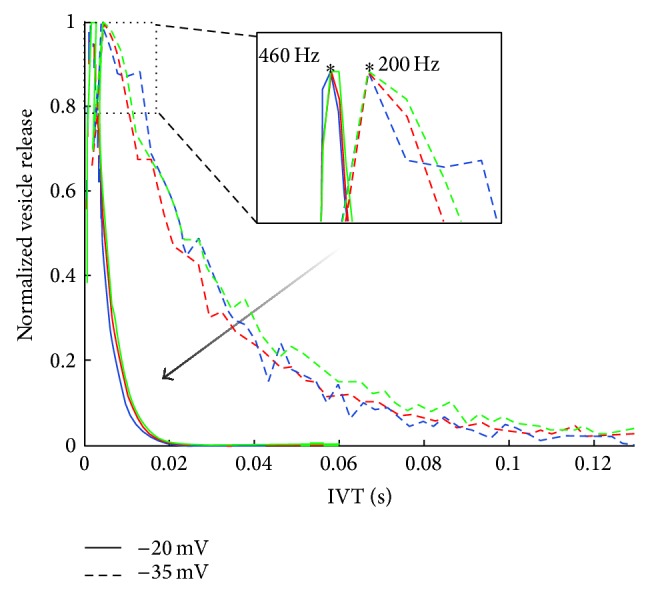
A line histogram of inter-vesicle release time intervals (IVT) at two different voltage levels for all three ribbon shapes shown in Figure 11. The blue line indicates the disk shape in Figure 11(a), the red line indicates the ellipsoid in Figure 11(b), and the green line the sphere shape in Figure 11(c). Note that as the intracellular voltage increases the direction of the normalised histogram shifts as indicated by the arrow. Furthermore the peak rate of release shifts from an IVT equal to 5 ms, that is, 200 Hz, to an IVT equal to 2.2 ms, that is, roughly 460 Hz, as the intracellular voltage increases from −35 mV to −20 mV.

As the intracellular voltage increases, the calcium ion concentration increases via a more frequent opening and closing of calcium channels. As is shown in Figure 4 the “open” time of the calcium channels is practically constant as voltage changes, and on the contrary the “close” time decreases. Essentially this means that as the intracellular voltage increases, the frequency of calcium channels opening increases but their open duration remains unchanged. This higher calcium channels opening frequency is indicated as a more potent release of vesicles and furthermore as a higher rate of release. Analysis results shown in Figure 12 explicitly show that the shape of the ribbon does not affect the rate of release, but instead the increasing intracellular voltage makes the rate of vesicle release faster. As is shown in Figure 12 the peak value of inter-vesicle release time intervals which indicates the vesicle release most frequent interval is becoming smaller as intracellular voltage becomes bigger.

### 5.2. Place Coding and Phase Locking

**Figure 14:**
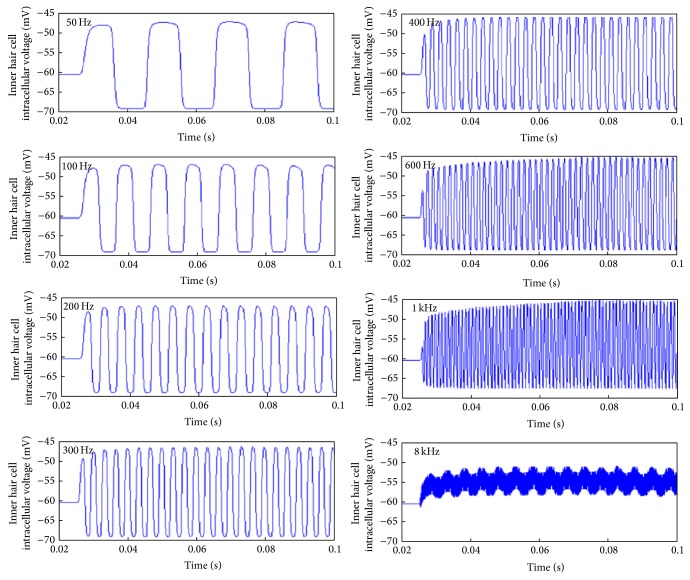
The resulting inner hair cell voltages from the MAP model following auditory stimulation with pure tone sounds at 80 dB SPL as recorded from the inner hair cell residing at the basilar membrane point with a best frequency corresponding to the stimulating pure tone. Note that only the first 100 ms is shown for clarity.

**Figure 15:**
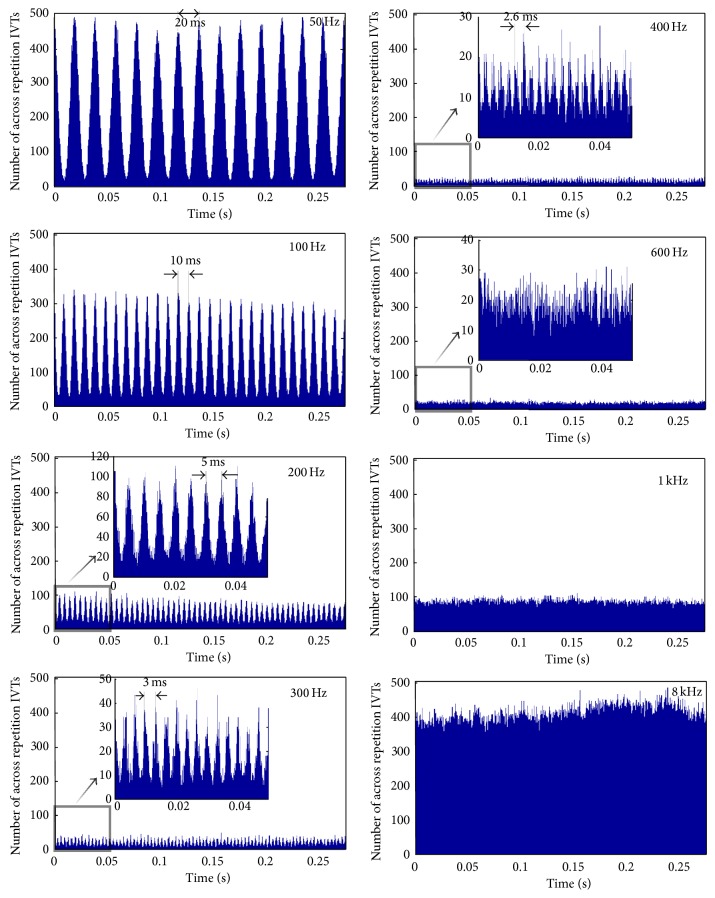
Across repetition inter-vesicle release time intervals for various pure tone sounds. The stimulating voltages caused then are shown in Figure 14. The method used to generate the histograms is shown in Figure 13.

**Figure 16:**
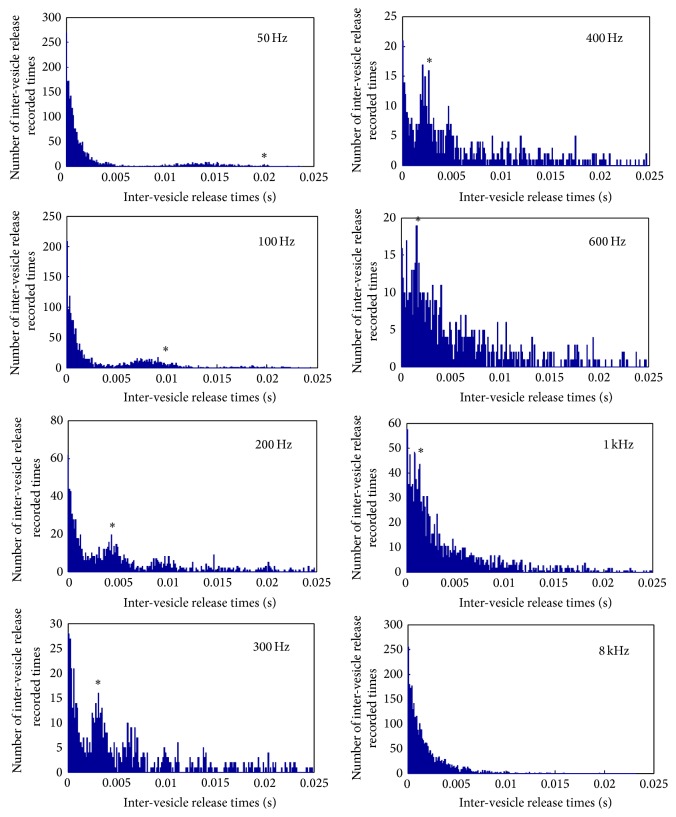
Inter-vesicle release time intervals (IVT), or period histograms for vesicle release for various pure tone sounds. The stimulating voltages caused then are shown in Figure 14. The asterisk on the plot indicates the IVT that corresponds to the frequency of the stimulating pure tone; note that the frequency of release is equal to the reciprocal of the IVT.

The model developed has amalgamated human auditory periphery with a mammalian model of the auditory synapse due to a lack of electrophysiological measurements of the human auditory synapse. A collection of results describing the temporal behaviour of the neurotransmitter vesicle release for a given spatial location on the basilar membrane is shown in Figures 14–16. The analysis performed aimed to provide insight into the phase locking or place coding nature of vesicle release and assess their effects at the synapse level.

**Figure 17:**
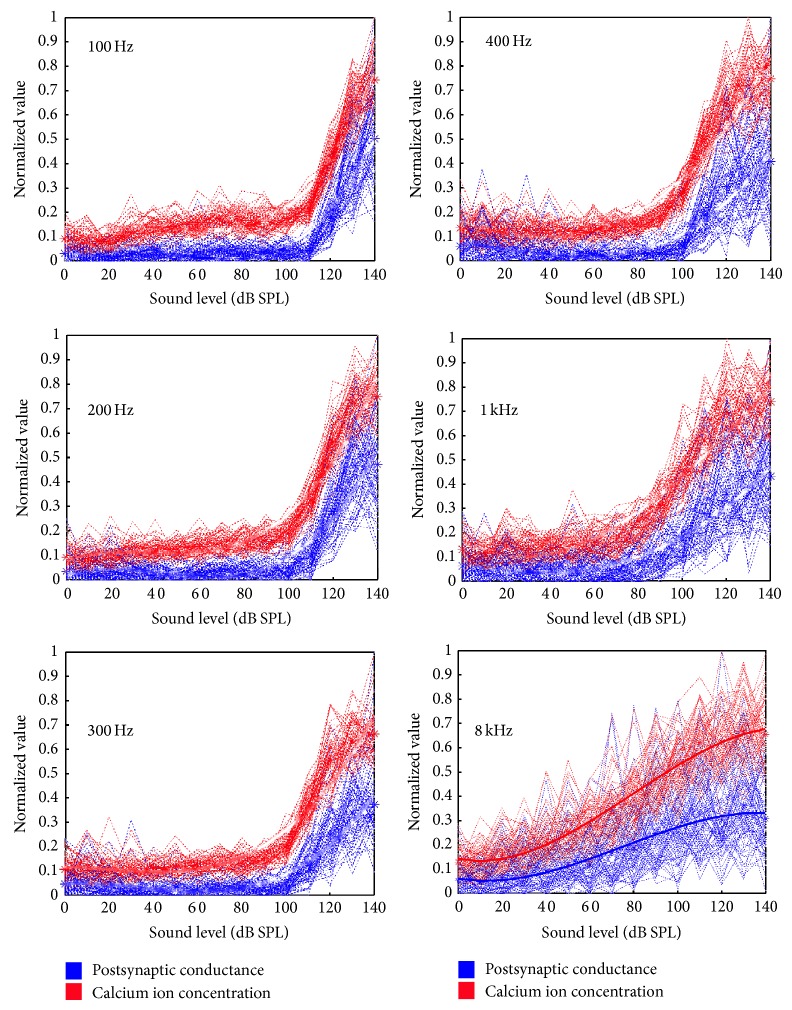
The effects of varying intensity (in dB SPL) of an input acoustic wave are investigated. Sound snippets, 200 ms in duration, were generated. Their frequencies varied: 100 Hz, 200 Hz, 300 Hz, 400 Hz, 1 kHz, and 8 kHz, all recorded at the corresponding best frequency position on the basilar membrane. The intensity of each sound snippet was increased in gradual steps of 10 dB SPL from 0 dB SPL to 140 dB SPL. Note that a “best fit” line is drawn for the 8 kHz plot because of the great variance in the model outputs.

The main trends observed are as follows: (1) this first is a reduction in vesicle release rate as frequency increases from 50 Hz to 400 Hz–600 Hz, followed by an increase in vesicle release rate as the frequency continues to increase to 8 kHz (see Figure 15),  (2) The second is an increase in vesicle release phase locking abilities as frequency increases from 50 Hz to 400 Hz–600 Hz, followed by a decrease in vesicle release phase locking as the frequency continues to increase to 8 kHz (see Figure 16),  (3) and as the sound frequency increases above 400 Hz, both the postsynaptic conductance and the presynaptic current become more linearly responsive to increasing sound levels. For the lower frequencies the response is relatively flat with an exponential rise for sounds above 100 dB SPL (see Figure 17). A more in depth discussion on the role of the outer hair cells efferent system and modelling their compressive effects using the MAP model can be found elsewhere [45, 46].

## 6. Discussion

The model responses to sound stimuli indicate that there is no specific limit of determining where place coding starts but instead, as the temporal phase locking of the vesicle release deteriorates for an increasing value of the input frequency, a sigmoid-like postsynaptic conductance is observed. Another interesting observation in the release of synaptic vesicles is that subharmonics seem to be emphasised for lower frequencies. This result suggests that the synaptic mechanism when encoding a phase-locked sound frequency will release a distribution of vesicles with a time gap around the time period of the resonant frequency but will also release vesicle distributions with twice, three times the period, and so forth. This may be caused by vesicles that have already a few calcium ions bound to them but do not release on the current signal peak, which makes them very prone to release on the next signal peak in a much more synchronised manner.

The highest frequency where there is evidence of phase locking in the release of neurotransmitter vesicles is up to 300–400 Hz; see Figure 16. For higher frequencies it was observed that no discernible pattern could be extracted from the release of neurotransmitter vesicles but instead a linear relation was observed between the change of postsynaptic conductance of the auditory neurite and the increasing sound levels in dB SPL. This finding supports place coding for higher frequencies as it reveals a biological mechanism to estimate the spectral energy content of the incoming sound, through the graded response of the intracellular voltage. In other words the linearised response of the postsynaptic conductance, for high frequencies, allows the estimation of the frequency specific loudness.

A highly tunable model of the auditory periphery has been presented. The attempt to maintain correspondence between model and physiology has resulted into a large number of parameters. Such a feature is usually undesired in a model since it makes it too complex to analyse and understand its behaviour. In the presented model though all the parameters have been measured and reported by independent studies. Moreover, every stage of the presented model has been checked against expected physiological behaviour. Modelling both the immature and the mature active zone was necessary since much of the information stemming from various studies and experiments considers the maturity and transition of the synaptic ribbon from a prehearing to a hearing state. It was considered necessary during the development of the model to verify whether the transition between the immature and mature mode of operation of the model (see Figure 2) could also replicate the experimentally observed behaviour.

The new synapse model is designed with the knowledge that it will be used as a module in a more general computational tool which will be able to have its parameters and functionality upgraded modularly and conveniently as more experimental data is generated by new studies. The synaptic ribbon used in modelling the various responses shown in this paper corresponds to the morphology and the reported dynamics of a mammalian synapse, notably studies performed on mice and some morphological studies on human inner hair cells [3, 4, 47]. There is a lack of detailed experimental studies conducted by modern methods on human cells, a necessary condition to accurately tune our synapse model to a human auditory synapse. However, the generic use of the same biological mechanism through various species permits the assumption that the human ribbon synapse will not differ radically from other mammals.

The assumptions the model makes are that AMPA channels do not saturate and that no losses of glutamate exist in the synaptic cleft [41]. The nanodomain coupling that is presumed to exist between the calcium channels and the vesicular sensors in the auditory synapse [16, 30] excludes local calcium induced calcium release mechanisms (like ones found in muscles) and the possibility of coupled oscillation of calcium channels (i.e., no propagating waves exist) [35]. Therefore, a further assumption made in the spatial integration of the overall action of calcium channels is that there is no cooperation between calcium channels. This allows linear summation of the individual channel contributions when calculating the intracellular calcium ion concentration level [31].

Since the stochastic nature of vesicle release is important in formulating a statistical representation of the stimulus, every nondeterministic stage of the vesicle release paradigm was modelled by means of Monte Carlo stochastic models whose probabilities of state transitions were calculated based on kinetic schemes of macro-scale measurements, as explained in Section 2. Since the Monte Carlo simulation technique is notorious for its failing accuracy if the sampling rate is too low, all the signals processed with Monte Carlo techniques have been resampled to confirm the accurate estimation of state transitions [27].

The morphofunctional circumferential gradient at the basolateral area of the inner hair cell and the tonotopic tuning of the auditory synapse has not been taken into account when modelling the responses in Figures 14–16. It is not the scope of the presented model to tune synapses within a single inner hair cell to differentiate between high spontaneous and low spontaneous rate which can be elicited by the change of the synaptic ribbon [48]. This will be considered in future investigations.

Given the unclear mechanisms of the vesicular tethering and trafficking [13] the model based a compound fusion mechanism on the conjecture that the larger the fused vesicle becomes the easier it will be to fuse even more vesicles onto it simply because its surface area will become greater, until a limit is reached where the compound vesicle is large enough to occupy its maximum supported surface area on the synaptic ribbon. The fact that it captures the onset response and shows evidence of recovery of response after adaptation in forward masking tests (see Figure 8) gives indications that it might be a valid conjecture. Nonetheless for the sake of brevity this mechanism will be further tested and scrutinised in a future study.

The increasing size of the synaptic ribbon makes the release of vesicles more potent as is indicated in Figure 11 which follows from the fact that many more vesicles are held close to the presynaptic membrane therefore increasing the “ready for release” pool of vesicles. A hypothesis raised by the results (see Figure 12) is that the rate that vesicles are released seems to be unaffected by the synaptic ribbon shape given that the calcium channel density is optimal for the given shape to ensure adequate colocalization between “ready for release” pool of vesicles and calcium channels. However there is not enough evidence in this study to adequately describe what is an optimal calcium channel density for each ribbon morphology.

Figure 11(a) reports the structure of a mammalian photoreceptor and the virtual organelles use dynamics identical to the rest of the auditory relevant morphologies. Even though both auditory and photoreceptor calcium channels are of the L-type, the inner hair cell contains Ca_V_^2+^ 1.3 whereas the photoreceptor cell contains Ca_V_^2+^ 1.4 calcium channels [49]. This means that without further investigation, outcomes of this study cannot be assigned as photoreceptive behaviour.

## 7. Conclusion

This effort aims at closing the gap between isolated studies on the auditory synapse and models that describe the actions of the auditory periphery. Such progress allows for novel investigations with regard to the behaviour of the auditory synapse at various positions on the basilar membrane under physiologically relevant stimuli, for example, audio waveforms of varying intensity. The results presented in this paper shed some light on two aspects of sound encoding at the level of the inner hair cell synapse. First, the effect of changing the morphology of the synaptic ribbon is assessed and it was found that the dynamics of the system are affected by the ribbon shape, by increasing the rate of vesicle release. Second, the relative contributions of phase locking or place coding as a means to encode frequency and intensity of the acoustic waves have been examined. It has been found that there is a continuous transition from phase locking at low frequencies to place coding for higher frequencies. Furthermore for low intensity sound, phase locking of synaptic vesicle release seems to happen at the subharmonics of the fundamental.

Each inner hair cell is innervated by 10 to 30 auditory afferent neurons which although individually limited in their maximum spiking frequency together display patterns phase-locked with the sound for up to a few kHz. The subharmonic preference of vesicle release shown in the results presented here (see Figure 16) can aid in explaining this effect. As observed from the state transitions of the bound calcium ions to the sensor mechanism of the synapse (see Figure 9) fast fluctuations of voltage, a characteristic of higher frequencies, causes an incomplete filling of the five possible binding sites shown in (4). Subsequently the dissociation of the calcium ions from the “calcium sensor” is slow enough that when the next peak of the voltage arrives the release of the vesicle is greatly facilitated by the almost full “calcium sensor.” This essentially forms a leaky integration mechanism similar to action potential generation, but in this case the triggered event is the release of a vesicle. In other words when the stimulating signal period is too short, that is, high frequency oscillations, the following periods will provide enough calcium ions to release the vesicle, assuming the stimulating duration is long enough to contain multiple periods.

This mechanism of release may explain the phase locking observed at the collection of auditory nerve fibres at frequencies higher than the maximum frequency allowable by the refractory period of the auditory nerve [50]. If the stimulating frequency is higher than the inverse of the auditory nerve's refractory period the action potentials elicited will miss some peaks of the stimulus. The subharmonic vesicle release observed in Figure 16 agrees with this effect, and furthermore the appearance of discrete peaks of subharmonic vesicle release suggests quantal and discrete phase shifts of the vesicle release events, which when their effects are summed across the entire cochlear nerve will provide phase locking effects as shown in previous studies [51].

A function for the synaptic ribbon that is suggested by the model outputs (see Figure 8) is that the purpose of tethering vesicles onto the ribbons surface is not to convey them to the presynaptic membrane but instead to keep the vesicles close enough so as to fuse. This permits multivesicular release from the compound fused vesicles and is suggested to be a way for the ribbon to encode signal onsets in a calcium ion independent way, as there is no evidence that calcium ions aid in fusing of vesicles. If this is indeed the case then it is important because it can hypothetically permit the auditory synapse to encode time and intensity separately; time can be encoded via the onset detection attribute given by the compound fusion and intensity can be encoded by the ongoing activity of the calcium channels on the presynaptic membrane in response to the change in intracellular voltage.

## Figures and Tables

**Figure 13:**
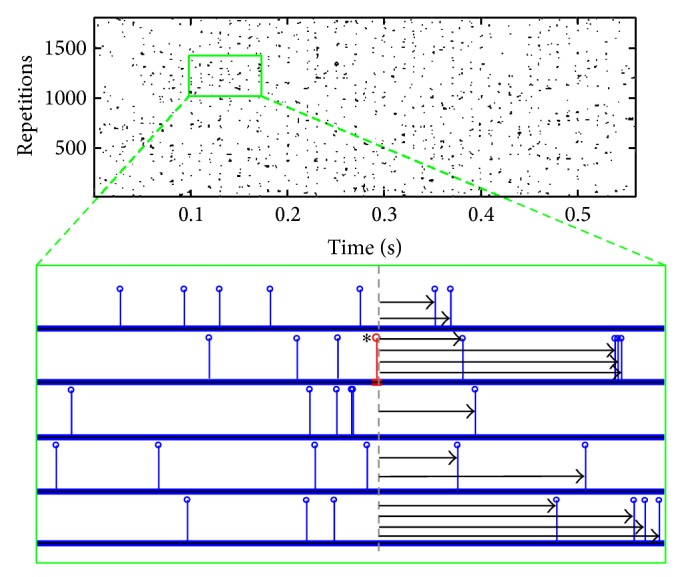
Illustration of the method used to estimate the across repetition inter-vesicle release time intervals. Each vesicle release event is taken individually, as the example event shown in red with an asterisk, and the time intervals from the given event to all the forward events are recorded, as exemplified by the arrows. Note the top raster plot shows the vesicle release events for auditory stimulation with a pure tone of 100 Hz, at 80 dB SPL as recorded from the inner hair cell residing at the basilar membrane point with a best frequency of 103 Hz. The method is very similar to the shuffled autocorrelogram described elsewhere [58, 59].

**Table 1:** Synapse morphology values.

Dimensions of the ribbon synapse	Value
Active zone radius^a^	300 nm [20, 52]
Number of calcium channels	90 ±^b^ 10 [43, 52, 53]
Calcium channels diameter	5 nm
Calcium channels spread^c^	Gaussian or uniform
Ribbon (disk) [α, β, γ]^d^	[40 nm, 166 nm, 127 nm]
Ribbon (ellipsoid) [α, β, γ]^d^	[100 nm, 166 nm, 127 nm]
Ribbon (sphere) [α, β, γ]^d^	[200 nm, 200 nm, 200 nm]
Membrane-ribbon distance, δ	35 nm
Vesicles diameter	35 nm with a S.D. of 10 nm [9]
Membrane-vesicles bond distance	15 nm [24]
Free vesicle density	3000 vesicles μm^−3^

^a^The radius describes the maximum distance possible for a calcium channel to exist from the centre of the simulation volume.

^b^The variation in channel number is drawn from a uniform distribution.

^c^This parameter controls the distribution pattern of the random allocation of channels from the centre of the synaptic ribbon as shown in Figure 2.

^d^These dimensions describe a triaxial ellipsoid as shown in Figure 3. The first set describes disk shape ellipsoids found in photoreceptors [42]. The second set corresponds to the dimensions of an 8-week mouse [54] and the last set to a sphere shaped ellipsoid of an amphibian papilla [54].

**Table 2:** Calcium buffer parameters and values.

Buffer^a^		Values^b^ [31]		
	K_D_ [μM]	k_*on*⁡_ [M^−1^s^−1^]	D [μm^2^s^−1^)]	Conc. [μM]
Endogenous	50	1 × 10^8^	15	0.5
EGTA	0.18	2.5 × 10^6^	220	2
BAPTA	0.22	4 × 10^8^	220	2
ATP	2300	5 × 10^8^	220	2

^a^The buffered ion species is Ca^2+^ which is assumed to have a diffusion coefficient of 220 μm^2^s^−1^ and an external concentration of 1.3 mM [55].

^b^K_D_ = dissociation constant, k_*on*⁡_ = forward reaction rate, D = diffusion coefficient, and Conc. = total buffer concentration (bound and unbound).
